# Stewart-Treves Syndrome as a Rare and Fatal Complication of Post-Traumatic Lymphedema on the Lower Extremity

**DOI:** 10.5826/dpc.1102a04

**Published:** 2021-03-08

**Authors:** Leandro Linhares Leite, Valeria Rossato

**Affiliations:** 1 Hospital São Lucas da PUCRS, Porto Alegre, Brazil

**Keywords:** Stewart-Treves syndrome, lymphedema, lymphangiosarcoma, hemangiosarcoma, lower extremity

## Introduction

Stewart-Treves syndrome (STS) is a rare disorder characterized by the development of angiosarcoma in an area of chronic lymphedema [1]. It was classically described in the upper limb after radical mastectomy with axillary lymph node resection, but there are reports related to idiopathic, congenital, post-surgical, traumatic or infectious lymphedema [1]. Only 10% of cases are reported in places other than upper limbs [1].

## Case Presentation

**Figure 1:**
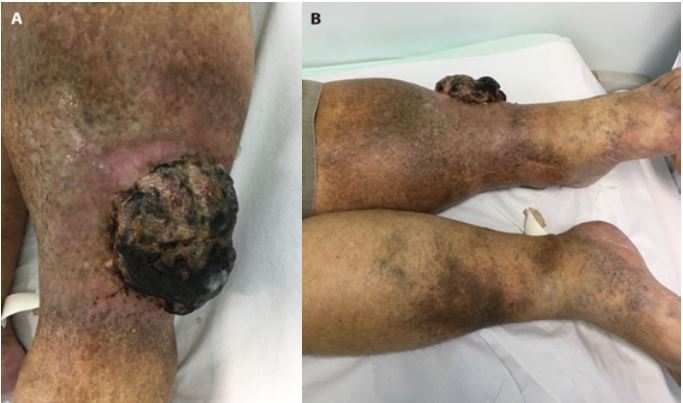
Clinical presentation of the lesion. (A) A vegetating and friable mass on the lateral aspect of the left leg. (B) The notable asymmetry between the legs caused by lymphedema in the left leg. Note that there is also edema due to chronic venous insufficiency in both legs.

A 70-year-old man presented with a 2-year history of a vegetating friable mass with progressive growth on his left leg and mild local pain (Figure 1A and B).

**Figure 2:**
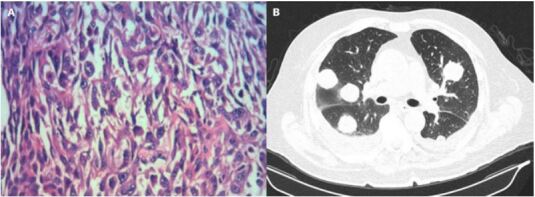
Images of complementary studies of the case. (A) Spindle cells with nuclear atypia and prominent nucleoli varying in size and shape. H&E, original magnification ×100. (B) Multiple pulmonary nodules with the compatible appearance of metastatic implants.

He had a history of a car accident with trauma to the left thigh 21 years before that complicated with chronic lymphedema in the affected limb. Incisional biopsy showed dermal vascular proliferation with positive immunohistochemistry (IHC) for CD31, CD34 and factor VIII, a condition compatible with angiosarcoma. The patient was referred for surgical excision, and the histopathological analysis of the specimen revealed high-grade undifferentiated malignancy, fusocellular and epithelioid cells with significant nuclear atypia, areas of necrosis, and fibromyxoid stroma (Figure 2A).

IHC was positive for CD31, FLI-1, and factor VIII and negative for human herpesvirus-8. Computed tomography revealed lung metastases (Figure 2B), and the patient died of complications from pulmonary hemorrhage 3 weeks after surgery.

## Conclusions

Cutaneous angiosarcomas are aggressive tumors that emerge from the vascular endothelium, usually related to predisposing factors such as chronic lymphedema and radiation exposure [1]. SST was first described in 1948 as a cutaneous lymphangiosarcoma in a series of 6 cases associated with chronic lymphedema after radical mastectomy for breast cancer. Angiosarcomas usually take 5 to 15 years to arise after mastectomy [1]. It is postulated that lymphedema creates an environment of local immunosuppression and impairs immune surveillance mechanisms, predisposing one to the emergence of atypical angiogenesis and malignancy [2]. Immunohistochemistry is often necessary to confirm the diagnosis and tissue origin and stain positive for factor VIII, CD34, CD31, and vimentin. The main differential diagnosis is Kaposi sarcoma, which is usually identified by immunohistochemistry analysis for human herpesvirus 8 [1]. The prognosis is poor, given the high rate of local recurrence and tendency for early metastases, mainly to the lungs [1].

This extremely rare case presented as a growing mass located on the lower limb with no previous history of lymphatic surgery, radiation, or malignancy. The late diagnosis contributed to the bad outcome, as the best management for STS is early diagnosis and surgical removal with amputation or wide local excision [1]. Adjuvant treatment with chemotherapy and radiotherapy is usually recommended, but their contributions remain uncertain [1]. Despite treatment, the prognosis is still poor with a mean survival time of 19 to 34 months, and the majority of the patients die from metastatic disease within 2 years [1].

Notwithstanding the aggressiveness, this tumor develops in a predictable and specific clinical context with well-established predisposing factors. Thus, physicians should be alert of new or growing lesions in lymphedema sites, regardless of location, to provide early diagnosis and increase the chances for successful treatment. Furthermore, preventive methods to treat lymphedema, such as weight loss, physiotherapy, and compressive measures, should be encouraged [1].
